# Management and Outcomes of Paediatric Intracranial Suppurations in Low- and Middle-Income Countries: A Scoping Review

**DOI:** 10.3389/fsurg.2021.690895

**Published:** 2021-08-12

**Authors:** Setthasorn Zhi Yang Ooi, Dawin Sichimba, David Ulrich Dalle, George Higginbotham, Berjo Dongmo Takoutsing, Nourou Dine Adeniran Bankole, Abdullah Egiz, Jay Kotecha, Rosaline de Koning, Stéphane Nguembu, Yvan Zolo, Yao Christian Hugues Dokponou, Samuel Chilawa, Soham Bandyopadhyay, Ulrick Sidney Kanmounye

**Affiliations:** Research Department, Association of Future African Neurosurgeons, Yaounde, Cameroon

**Keywords:** management, outcomes, intracranial, suppurations, paediatric, infection, low and middle income countries

## Abstract

**Introduction:** Intracranial suppurations account for a significant proportion of intracranial masses in low- and middle-income countries (LMICs), particularly among children. The development of better imaging equipment, antibiotics, and surgical techniques has enabled significant progress in detecting and treating intracranial abscesses. However, it is unclear whether these advances are accessible and utilised by LMICs. In this review, we aimed to describe the landscape of paediatric intracranial suppurations in LMICs.

**Methods:** This scoping review was conducted using the Arksey and O'Malley framework. MEDLINE, EMBASE, WHO Global Index Medicus, AJOL and Google scholar were searched for relevant articles from database inception to January 18th, 2021. Publications in English and French were included.

**Results:** Of the 1,011 records identified, 75 were included. The studies, on average, included 18.8 (95% CI = 8.4–29.1) children (mean age: 8.2 years). Most children were male (62.2%, 95% CI = 28.7–95.7%). Intracranial suppurations were most commonly (46.5%) located in the supratentorial brain parenchyma. The most prevalent causative mechanism was otitis (37.4%) with streptococcus species being the most common causative organism (19.4%). CT scan (71.2%) was most commonly used as a diagnostic tool and antibiotics were given to all patients. Symptoms resolved in 23.7% and improved in 15.3% of patients. The morbidity rate was 6.9%, 18.8% of patients were readmitted, and the mortality rate was 11.0%.

**Conclusion:** Most intracranial suppurations were complications of preventable infections and despite MRI being the gold standard for detecting intracranial suppurations, CT scans were mostly used in LMICs. These differences are likely a consequence of inequities in healthcare and have resulted in a high mortality rate in LMICs.

## Introduction

Intracranial suppurations are infections of the central nervous system (CNS) characterised by the production and the accumulation of pus within the brain parenchyma and meningeal spaces (1, 2). Intracranial suppurations commonly occur through infection of a neighbouring site spreading to the CNS, such as sinusitis, otitis, or mastoiditis (3). Other causes include direct trauma, surgery, or haematogenous spread (3). Intracranial suppurations are generally divided into three broad categories: brain abscesses, in which the infection is based within the brain parenchyma; subdural empyemas, where the pus sits between the dura and the arachnoid membrane; and extradural empyemas, where the pus accumulates between the dura and the skull (1, 3).

Intracranial suppurations can be common, with brain abscesses alone accounting for 8% of intracranial masses in low-and-middle-income countries (LMICs), compared to only 2% in high-income countries (HICs) (4). With a mortality rate of up to 25% (5), they represent a significant burden of disease in LMICs. One contributing factor to the higher incidence in LMICs is the rate of HIV infection in these countries, as immunosuppression is a significant risk factor for the development of intracranial suppurations (6). With an increasing number of HIV-positive patients presenting with intracranial suppurations in LMICs, elucidating the current ability of local healthcare systems to cope with these cases is key. One critical facet to tackling intracranial suppurations is identifying the causative organism. The causative organism behind intracranial suppurations vary, but are largely bacterial species; the most common causative organisms being streptococcus and staphylococcus species (7). The bacterial species causing the intracranial suppuration vary depending on the primary infection site, the patient's underlying health, geographical location, and the patient's age. Of note, children are particularly vulnerable to intracranial suppurations due to their susceptibility to the development of adjacent infections, such as otitis and sinusitis. In LMICs, children represent a large population demographic, accounting for nearly half the population in some countries (8). Additionally, the highest global incidence rate of otitis media has been reported in Sub-Saharan Africa and South Asia (9); the majority of cases occur in the paediatric age group (10).

While the development of better imaging equipment, antibiotics, and surgical techniques have enabled significant progress in our ability to detect and treat intracranial abscesses. These advances are not necessarily accessible in low-resource settings such as LMICs. For example, one paper suggests that only 27.3% of patients in Cameroon with intracranial abscesses have access to an MRI scan (11), despite this mode of imaging being recommended for diagnosis (12). Once intracranial suppuration is identified, medical treatment with antibiotics and surgical treatment aiming to evacuate the pus are recommended (13). However, it is still unclear to what extent LMICs have access to these recommended treatment methods.

Therefore, the research question of the present study is: how are paediatric intracranial suppurations managed in LMICs? Our review primarily aims to provide an overview of the epidemiology, management, and outcomes of this population of patients. We aim to detect heterogeneity in treatment and whether this treatment deviates from the gold standard across LMICs. Gaining an understanding of the current landscape in LMICs is a vital step toward ensuring efforts are being directed toward areas most in need, both geographically and in terms of aspect of care: diagnostics, pharmacological treatment, and surgical treatment.

## Methods

A scoping review on the epidemiology, management, and outcomes of paediatric intracranial suppurations in LMICs was conducted as per the published and registered protocol (14). The Arksey and O'Malley scoping review framework was used to guide the scoping review (15). The Preferred Reporting Items for Systematic Review and Meta-Analysis extension for Scoping Reviews (PRISMA-ScR) guidelines were used to report the findings (16).

### Inclusion and Exclusion Criteria

We included studies that fulfilled the specific inclusion criteria discussed in our published protocol. Studies of interest included studies that discussed intracranial paediatric (defined as between 0 and 18 years of age) suppurations in LMIC populations. We also included journal articles, reviews, case reports, and letters. There were no restrictions to the period of the publications considered to ensure that all relevant articles published from database inception to date of search are captured. Publications in English and French languages were considered. We excluded studies that (a) did not include paediatric populations (or did not have disaggregated data about a paediatric population), (b) did not discuss intracranial suppurations, (c) were neither written in English nor French, and (d) were not related to LMICs (or did not have disaggregated data about an LMIC population), (e) did not have accessible full-text, and (f) are conference abstracts.

### Search Strategy

The search protocol for this scoping review was executed in MEDLINE, EMBASE, WHO Global Index Medicus, and African Journals Online covering the period between database inception to January 18, 2021. The search strategy used variants and combinations of search terms related to children, intracranial suppurations, and LMICs. The Appendix in our published protocol shows the exact content and order of the search string queries (14). A hand search of Google Scholar was further conducted to identify additional articles that were not captured in the above process.

### Study Selection

All the articles resulting from the search were exported into Rayyan (17), where duplicates were identified and deleted. Rayyan is a professional research software that is widely used by collaborators for ease of study selection decisions. The study selection process consisted of multiple steps. Firstly, an online training session was organised to ensure all authors understood the pre-defined inclusion and exclusion criteria of the study. Next, two reviewers of SZYO, DS, DUD, GH, BDT, BNDA, AE, JK, RdK, SN, YZ, DYCH, SC, SB and USK independently screened the titles and abstracts of the identified articles based on the criteria. Any disagreement between the two reviewers' decisions prompted further discussion. If a disagreement persisted, a senior author (SB or USK) was sought to resolve the disagreement. The full texts of the remaining articles were retrieved and screened by two reviewers (of SZYO, DS, DUD, GH, BDT, BNDA, AE, DYCH and USK) independently.

### Data Extraction

Prior to data extraction, an Excel proforma sheet was used to ensure all participants in the data extraction step were extracting data homogeneously. The Excel sheet included columns of specific interest for data extraction such as study design, patient demographics/characteristics, type of intervention and outcomes of care. Data extraction was performed in two stages, a pilot stage followed by a proper stage. The pilot stage consisted of having multiple authors, each going through the same 10 selected articles to extract data. This was to ensure that all participant authors were able to extract data accurately for a swift data analysis stage. It was also important to pilot this stage to ensure the data collection sheet was reflective of the included studies.

### Data Analysis

Extracted data was analysed by SPSS v.26 (IBM, USA). Pooled statistics were calculated using measures of central tendency and spread.

## Results

**Figure 1:**
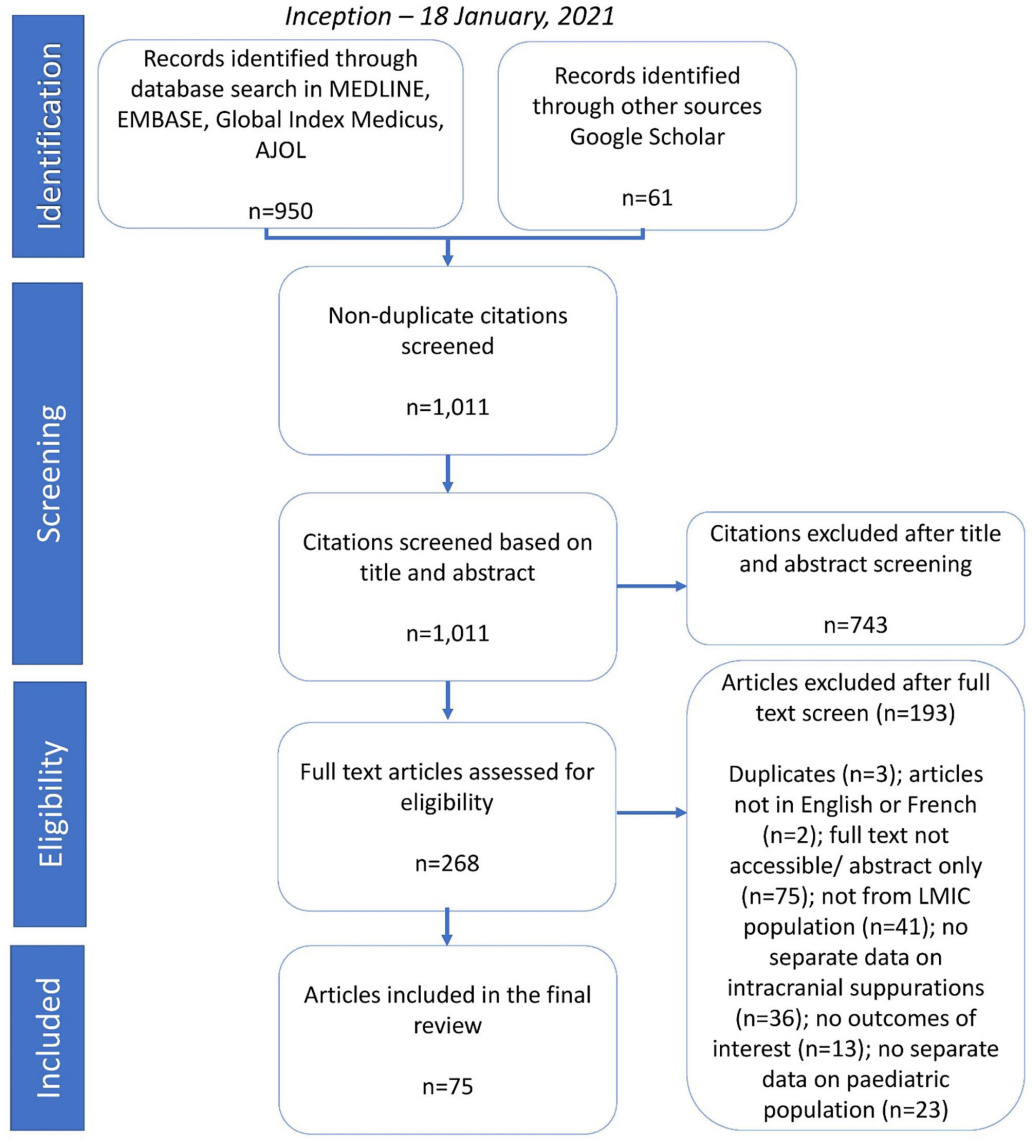
PRISMA flow chart.

We identified 1,011 records: 950 (93.97%) via the database search and 61 (3.0%) *via* supplemental hand search. We excluded 743 articles (73.5%) at title and abstract screening, and 193 (19.1%) at full text screening. Seventy-five articles (7.4%) were eligible for inclusion (Figure 1).

Thirty-three studies (44.0%) reported on the management and outcomes of paediatric suppurations in India. The 2000–2009 decade saw the highest number of publications (*n* = 33, 44.0%) and the majority of studies were case reports (*n* = 40, 53.3%) (Table 1). The study populations consisted of 18.8 (95% CI = 8.4–29.1) children on average. The children were 8.2 (95% CI = 6.5–10.0) years old and most were male (mean = 11.7, 62.2%, 95% CI = 28.7–95.7%).

**Table 1:** Characteristics of the 75 studies included in the review.

**Characteristic**	**Frequency (percentage)**
**Study setting**	
India	33 (44.0)
Pakistan	7 (9.3)
Malaysia	6 (8.0)
Iran	5 (6.7)
Nigeria	4 (5.3)
Turkey	4 (5.3)
Thailand	2 (2.7)
Benin	1 (1.3)
Brazil	1 (1.3)
Cameroon	1 (1.3)
China	1 (1.3)
Egypt	1 (1.3)
Gabon	1 (1.3)
Indonesia	1 (1.3)
Malawi	1 (1.3)
Nepal	1 (1.3)
Senegal	1 (1.3)
South Africa	1 (1.3)
South Korea	1 (1.3)
Tunisia	1 (1.3)
Zambia	1 (1.3)
**Year of publication**	
1970–1979	2 (2.7)
1980–1989	2 (2.7)
1990–1999	6 (8.0)
2000–2009	33 (44.0)
2010–2019	27 (36.0)
2020	1 (1.3)
**Study design**	
Case report	40 (53.3)
Cross-sectional	18 (24.0)
Case series	9 (12.0)
Cohort	7 (9.3)
Letter to the editor	1 (1.3)

**Figure 2:**
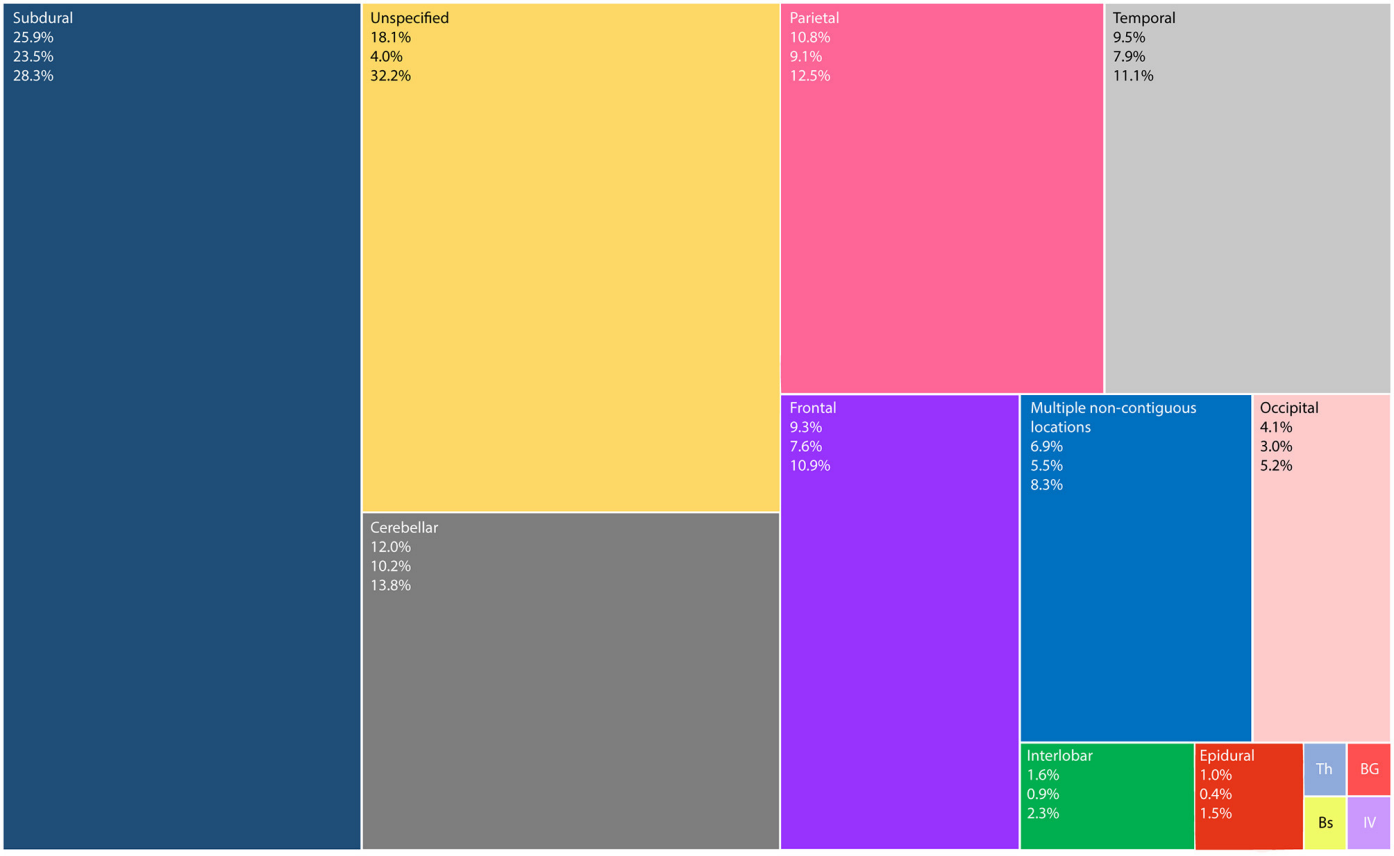
TreeMap showing the distribution of intracranial suppurations (words at the top left corner), their mean percentage (first number), lower and upper limits of the 95% confidence interval (second and third numbers, respectively). Th—Thalamic 0.2% (95% CI = 0.0 to 0.5%); BG—Basal Ganglia 0.2% (95% CI = −0.1 to 0.4%); Bs—Brainstem 0.2% (95% CI = −0.1 to 0.4%); and IV—Intraventricular 0.2% (95%CI = −0.1 to 0.4%). Unspecified corresponds to supratentorial intra-axial lesions, whose locations were not further clarified.

The majority of cases were intraparenchymal abscess. Intracranial suppurations were located in supratentorial intra-axial (46.5%, 95% CI = 43.7–49.3%), subdural (25.9%, 95% CI = 23.5–28.3%), infratentorial intra-axial (22.3%, 95% CI = 20.0–24.6%), and epidural (1.0%, 95% CI = 0.4–1.5%) spaces. Figure 2 shows the distribution of intracranial suppurations by location within the brain.

**Figure 3:**
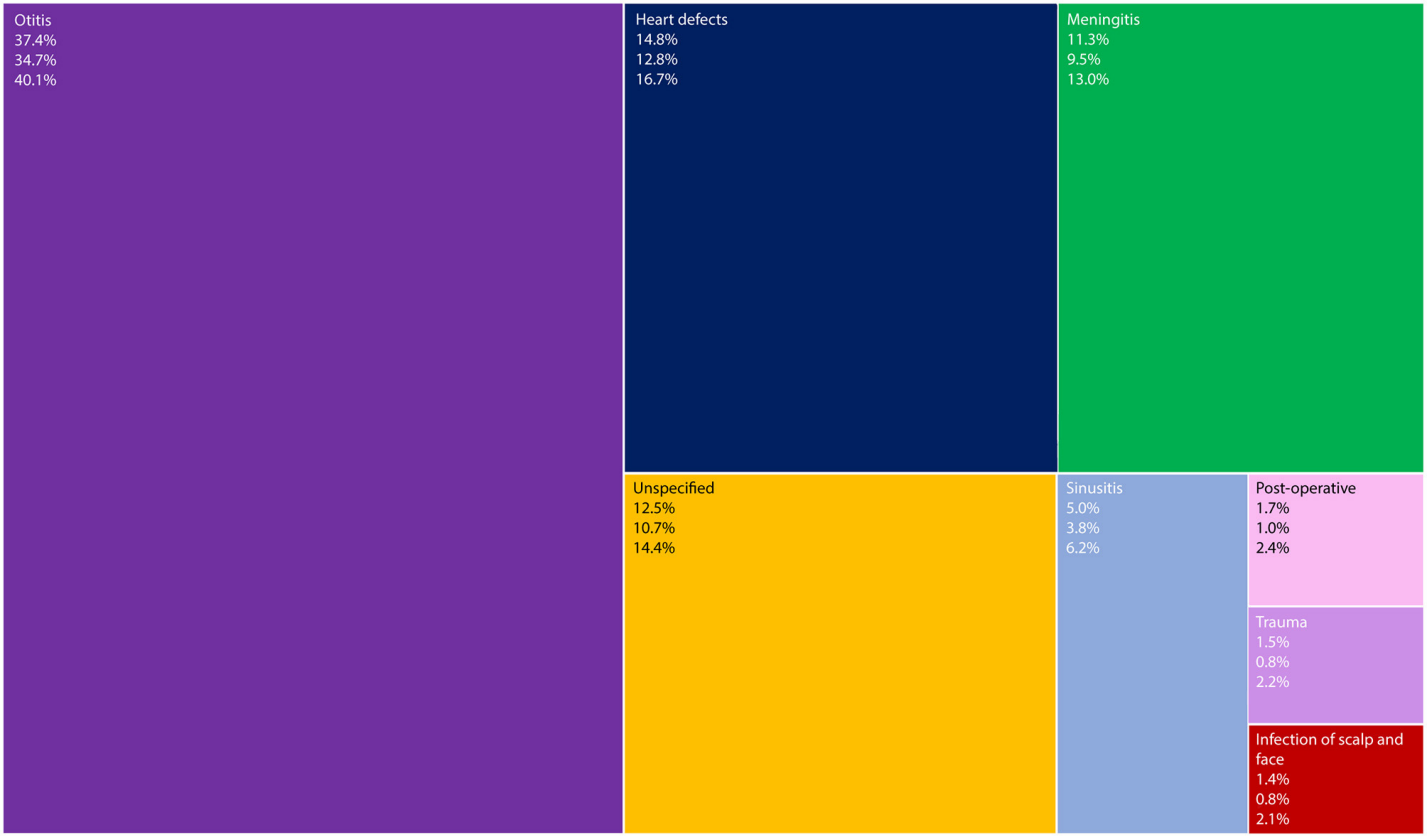
TreeMap showing causative mechanisms of intracranial suppurations (words at the top left corner), their mean percentage (first number), lower and upper limits of the 95% confidence interval (second and third numbers, respectively).

The most prevalent causative mechanism was otitis (37.4%, 95% CI = 34.7–40.1%), followed by heart defects (14.8%, 95% CI = 12.8–16.7%) (Figure 3).

Streptococcus species were the most common causative organisms (19.4%, 95% CI = 17.2–21.6%) and 12.8% (95% CI = 10.9–14.6%) of cultures were negative. 5.5% (4.2–6.7%) of intracranial suppurations were polymicrobial (Table 2). Data on the specific causative organisms responsible for 58 cases of subdural empyema and one case of epidural abscess was available (Appendix S1).

**Table 2:** Causative organisms of intracranial suppurations.

**Organisms**	**Percentage**	**Lower limit 95% confidence interval**	**Upper limit 95% confidence interval**
Streptococcus species	19.4%	17.2%	21.6%
Negative culture	13.1%	11.2%	15.0%
Polymicrobial	5.5%	4.2%	6.8%
Staphylococcus species	5.4%	4.2%	6.7%
Proteus species	4.9%	3.7%	6.1%
Haemophilus influenza	2.6%	1.7%	3.5%
Pseudomonas species	2.6%	1.7%	3.4%
Escherichia coli	1.5%	0.8%	2.2%
Bacteroides species	1.4%	0.8%	2.1%
Unspecified anaerobes	1.4%	0.8%	2.1%
Salmonella	1.4%	0.8%	2.1%
Klebsiella species	1.2%	0.6%	1.8%
Bacillus cereus	0.6%	0.1%	1.0%
Mycobacterium tuberculosis	0.5%	0.1%	0.9%
Citrobacter	0.5%	0.1%	0.9%
Peptostreptococci species	0.4%	0.0%	0.7%
Enterococcus	0.4%	0.0%	0.7%
Enterobacter species	0.3%	0.0%	0.6%
Providentia species	0.2%	0.0%	0.5%
Ps. Pyocyaneus	0.2%	−0.1%	0.4%
Morganella morgagni	0.1%	−0.1%	0.2%
Eikenella	0.1%	−0.1%	0.2%
Plasmodium falciparum	0.1%	−0.1%	0.2%
Mycobacterium fortuitum	0.1%	−0.1%	0.2%
Cladosporium bantianum	0.1%	−0.1%	0.2%
Haemophilus aphrophilus	0.1%	−0.1%	0.2%

The results show that computed tomography (CT) scan (71.2%, 95% CI = 68.7–73.7%) was more commonly used than magnetic resonance imaging (MRI) (8.9%, 95% CI = 7.3–10.5%). Antibiotics were the most common treatment offered (100%), followed by burr hole aspiration (47.4%, 95% CI = 44.6–50.1%). The most common antibiotics used were metronidazole (23.6%) and cephalosporins (20.4%) across all types of suppurations. For subdural suppurations, a greater number of cases were surgically managed *via* a burr hole (*n* = 71) than a craniotomy (*n* = 50). The study also reports a mortality rate of 11.0% (95% CI = 9.3–12.8%) (Table 3).

**Table 3:** Management and outcomes of intracranial suppurations.

**Management and outcomes**	**Percentage**	**Lower limit 95% confidence interval**	**Upper limit 95% confidence interval**
**Neuroimaging**			
CT scan	71.2%	68.7%	73.7%
MRI	8.9%	7.3%	10.5%
**Treatment**			
Antibiotics	100%		
Burr hole aspiration	47.1%	44.3%	49.9%
Craniotomy	34.7%	32.1%	37.3%
Insertion of drain	10.3%	8.6%	12.0%
Mastoidectomy	5.5%	4.2%	6.8%
Subdural paracentesis	1.5%	0.8%	2.2%
Shunt	1.2%	0.5%	1.8%
Bone flap removal	0.1%	−0.1%	0.2%
Craniectomy	0.1%	−0.1%	0.2%
**Outcomes**			
Neuro-intensive care admission rate	3.3%	2.4%	4.3%
Symptoms resolved	23.7%	21.3%	26.0%
Symptoms improved	15.3%	13.3%	17.3%
Symptoms unchanged	2.2%	1.4%	3.1%
Symptoms worsened	4.7%	3.5%	5.9%
Mortality rate	11.0%	9.3%	12.8%
Readmission rate	18.8%	16.7%	21.0%

## Discussion

### Key Findings

This scoping review is the first to describe the epidemiology, management, and outcomes of paediatric intracranial suppurations across LMICs. The average age of the included children were 8.2 years, with a male predominance. Most of the intracranial suppurations had a supratentorial intra-axial location with the most prevalent causative mechanism being otitis. Streptococcus species were the most common causative organisms isolated on positive cultures, however, a few positive cultures yielded polymicrobial growths. CT scan was the neuroimaging technique for diagnosis and follow-up in the majority of studies. All patients received antibiotics. Where surgical management was required, the most common approach used was burr hole aspiration, followed by craniotomy. Resolution of symptoms was the most frequent outcome pattern after treatment; however, there was a considerable rate of readmission and a low but significant mortality rate.

### Implications

The pattern of location of intracranial suppurations across LMICs found in this review is similar to reports from single countries (18, 19), suggesting that our scoping review is representative of the international picture and has not been biased by reports from single centres or articles focusing on a particular location of intracranial suppurations. However, otitis followed by heart defects were the most common predisposing factor in our study. This is contradictory to a national report of intracranial suppurations conducted by Ozsurekci et al. in 2012 in Turkey, who reported congenital cyanotic heart disease to be the most common predisposing factor (33.3%) with otogenic infections and meningitis accounting for just 16 and 8% respectively (19). This can be attributed to the introduction of the pneumococcal vaccine into the Turkish national immunisation schedule in 2008 and highlights a potential public health intervention that other LMICs may emulate to decrease the incidence of otogenic infections and intracranial suppurations in their country (19, 20). As of 2016, 59 countries did not have the pneumococcal vaccine in their childhood immunisation schemes. Access to vaccines in most LMICs has been limited by cost but this challenge has been addressed by GAVI, the Vaccine Alliance which has sponsored the pneumococcal vaccine in 54 countries (21, 22). Given otitis has been identified to be the most prevalent predisposing factor to intracranial suppurations across LMICs, it may also be possible to reduce intracranial suppurations by promptly treating otitis with effective antibiotics. However, clinicians should also be mindful to assess the efficacy of the antibiotic in treating otitis to prevent antibiotic overuse (23). It is currently best practice in the treatment of otitis for a clinician to reassess the patient 72 h after initiation of treatment to note any progress and if there is no significant improvement to change the antibiotic treatment (24).

An important finding is that despite MRI with contrast being the gold standard imaging technique in diagnosing and managing intracranial suppurations (12), CT scan was the neuroimaging technique used in 72.9% of cases. This is possibly due to reduced access to MRI neuroimaging in LMICs (11). All patients in our study received a course of antibiotic therapy, which is a positive sign for the movements that have cited the importance of access to antibiotics globally (25). However, it was unclear from the included texts whether the most appropriate antibiotics were being used in each case. A minority of cases did not have any surgical management. This may be because surgical management was not needed, due to limited access to neurosurgical centres, difficulty in surgical drainage for certain intracranial suppurations and/or lack of aseptic equipment in certain LMIC centres. There is a disparity between LMICs and HICs in terms of the surgical technique utilised: our findings suggest that LMICs primarily used burr hole aspiration, whilst existing reports from HICs suggest craniotomies have a greater prevalence (18). The lack of craniotomies conducted in LMICs may be accounted for by several reasons: (i) the difficulties in maintaining the higher levels of sterilisation required for that procedure in resource-limited environments (26); (ii) the longer operating times and higher re-operating rates typically associated with craniotomies (27); (iii) the higher perceived costs of craniotomies; and (iv) the difficulties in following up a patient after discharge. Given the lack of data around this topic, it is pertinent that a study is conducted to identify the relevant factors that influence the surgeons' decision to conduct a burr hole or a craniotomy in LMICs. The lack of access to gold-standard diagnostic tools and management may explain the high rate of mortality of intracranial suppurations in LMICs (11.0%), with similar studies in HICs reporting mortality rates of 3.2% (28).

Receiving adequate treatment at a facility is unlikely to be the only factor behind the overwhelming difference in mortality rates between LMICs and HICs with the “Three Delays Model” (29) also citing delays in (i) seeking neurosurgical care, and (ii) identifying and reaching an appropriate neurosurgical facility as further compounding issues. The lengths of these delays may be a product of socioeconomic and cultural factors, accessibility of neurosurgical facilities and the availability of treatment. A poor patient outcome is highly likely if any of these factors lead to the delay. However, various strategies have been established to tackle these delays in LMICs (29). In the context of our review, education and employment of parents are crucial (29–31). Educating the public on the importance of recognising the signs and symptoms and the repercussions of inaction or delayed action will be a key step to address the first delay. Employment plays a key role in addressing the first and second delays as the lack of financial means may influence: (i) the urgency to seek care due to concerns of insurance and health costs and (ii) the mobility of the parent and child as this is reliant on the mode of transport the family uses. The second and third delays can be tackled through the role of local governments and stakeholders. Providing affordable and accessible public transport and building safe roads to hospitals and clinics would be recommended as means to approach the issues of the second delay. As for the third delay, the standardisation of training programmes, organisation of visitor teaching programmes, and expansion of recruitment of healthcare professional recruitment may resolve issues such as variability in practice, lack of competence in the management of the disease and shortage of staff.

Another important implication for LMIC healthcare systems is that more than 1 in 20 intracranial suppurations were found to be caused by polymicrobial infections. Polymicrobial infections increase the cost of management, placing an additional burden on resource-limited settings (32). These infections have been found to lead to a longer length of stay in hospitals, prolonged intravenous antibiotic administration (33), the use of more costly antibiotics (32, 34, 35), and a wide variety of complications and sequelae (36). Given its prevalence, clinicians should consider the use of broad-spectrum antibiotics early in the management course as a strategy to prevent potential complications due to polymicrobial infections; albeit being cautious of *Clostridium difficile* infection.

### Limitations

Whilst a scoping review is purposefully extensive in breadth, the inclusion of multiple heterogenous evidence sources limits the comparisons that can be made between studies. Specifically, different methodological approaches were used across the studies, with a lack of uniformity in outcome reporting. Conclusions that can be drawn from this review are limited by the quality of evidence in the available literature; the majority of included articles were case reports, with few cohort studies, and no randomised controlled trials. Our review was also limited to articles in English and French, and so failed to capture data from any studies published in alternative languages. Whilst English is the most common language of publication for medical journal articles (37), this is not universal, especially in LMICs. Furthermore, a number of studies were excluded on the basis of the lack a separate analysis for paediatric and adult populations. Whilst adherence to strict exclusion criteria was necessary to maintain the relevance of data, it may have led to the omission of useful information. Despite these limitations, our study provides a plethora of novel and useful information that can guide relevant stakeholders as to which areas need to be tackled to reduce the burden of intracranial suppurations.

There is an urgent need for a multisectoral and multidimensional approach to effectively curb the burden of paediatric intracranial suppurations in LMICs (38). Stakeholders involved in reducing health inequalities and ameliorating the well-being of populations are vital for this mission (39). This mission falls in line with the sustainable development goals number 1, 3, 4 and 10, which are no poverty, good health and well-being, quality education and reduced inequalities, respectively (40). Tackling poverty is particularly key, as poverty promotes poor health practices, and inadequate health infrastructures promote factors leading to paediatric intracranial suppurations (41). Moreover, paediatric intracranial suppurations can keep patients away from school, which may lead to poor quality of education in this age group. Therefore, there are multiple reasons a well-coordinated action of all stakeholders is necessary to reduce the burden of paediatric intracranial suppurations (42, 43). Reducing inequalities will permit patients in LMICs to receive the appropriate care required (44).

## Conclusion

This scoping review provides an overview of the management and outcome of paediatric intracranial suppurations in LMICs. The intracranial distribution of suppurations matched prior literature, as did the predominance of *Streptococcus* species as the causative organism. CT was commonly used, but the use of the gold standard diagnostic imaging modality (MRI) was limited. As MRI scans are better than CT scans at diagnosing suppurations, the choice of investigations may have delayed diagnosis. We also found that burr holes are more commonly used compared to craniotomies. As craniotomies have reduced rates of reoperations and are more likely to definitively clear the intracranial suppuration, the surgical management choice may have delayed definitive treatment. Moreover, whilst all studies reported management with antibiotic therapy, surgical management was less prevalent than in prior literature. Delays of diagnosis and definitive treatment are known to increase morbidity and mortality rates; this may explain the 1 in 10 patients in our review who died, which is higher than reported in studies of populations in HICs, highlighting the necessity for improvements in care.

However, solving these issues may go beyond acquiring MRIs and encouraging surgeons to perform craniotomies. MRIs are expensive to maintain and may not be economically feasible to have. Craniotomies have increased infection risk and the lack of sterile fields may increase the rate of infections. Therefore, stakeholders in surgical care and the health of underserved populations should focus on tackling this area through strategies such as national vaccination programmes or the development of cheap sterile gowns. Lastly, as otitis is the most prevalent causative mechanism, early treatment with appropriate analgesia would be recommended as a preventative measure to developing intracranial suppurations. Timely interventions as such would cost less and lead to better patient outcomes than treating intracranial suppurations itself.

Future published literature regarding intracranial suppurations in LMICs should be encouraged to provide the anonymised dataset from which their data is based. Having access to this data would enable valuable disaggregated information, such as the management of different types of suppurations, to be extracted and combined with other studies focusing on the same research topics.

## Author Contributions

SO: conception, design, data extraction, data analysis, data curation, writing, reviewing and editing, and project administration. DS: conception, design, data extraction, writing, reviewing and editing, visual abstract. DD, BT, and GH: data extraction and writing. YD, SC, NB, and JK were involved in data extraction. AE: design, data extraction, writing, reviewing and editing. SN, YZ, and RK: writing. SB: conception, design, data extraction, writing, reviewing and editing. UK: conception, design, data analysis, writing, reviewing and editing. All authors contributed to the article and approved the submitted version.

## Conflict of Interest

The authors declare that the research was conducted in the absence of any commercial or financial relationships that could be construed as a potential conflict of interest.

## Publisher's Note

All claims expressed in this article are solely those of the authors and do not necessarily represent those of their affiliated organizations, or those of the publisher, the editors and the reviewers. Any product that may be evaluated in this article, or claim that may be made by its manufacturer, is not guaranteed or endorsed by the publisher.

## Appendix S1

**Table:**

**Microorganism**	**Subdural empyema**	**Epidural abscess**
Escherichia coli	4	1
Haemophilus influenzae	13	0
Pseudomonas species	5	0
Staphylococcus species	6	0
Streptococcus species	15	0
Klebsiella species	2	0
Anaerobes	3	0
Bacillus cereus	1	0
Plasmodium falciparum	1	0
Mycobacterium tuberculosis	1	0
Mycobacterium fortuitum	1	0
Ps. Pyocyaneus	2	0
Salmonella	1	0
Polymicrobial	3	0
